# Metabolic Dysfunction-Associated Steatotic Liver Disease (MASLD): New Perspectives on an Evolving Epidemic

**DOI:** 10.3390/jcm14248872

**Published:** 2025-12-15

**Authors:** Gerond Lake-Bakaar

**Affiliations:** 1 Beth Israel Deaconess Medical Center, Harvard Medical School, Boston, MA 02215, USA; glakebak@verizon.net; 2 Department of Medicine, Saint Anthony Hospital, Lakewood, CO 80228, USA

**Keywords:** nonalcoholic fatty liver disease (NAFLD), metabolic dysfunction-associated steatotic liver disease (MASLD), hepatic blood flow, hepatic artery buffer response (HABR), oxygen-nutrient mismatch

## Abstract

The absence of a unifying pathogenetic mechanism in metabolic dysfunction-associated steatotic liver disease (MASLD), formerly known as non-alcoholic fatty liver disease (NAFLD), has significantly hindered therapeutic progress. Appreciation that the delivery of excessive amounts of calories to the liver via the portal circulation might be a key parallel between MASLD and the twin steatotic liver disease, alcohol-related liver disease (ALD), establishes a consolidated framework that could guide rational drug design and precise therapeutic approaches. This review contends that, in both ALD and MASLD, the unique dual blood supply to the liver, from both portal vein and hepatic artery as well as the distinctive blood flow control physiology, prevents hepatic arterial oxygen delivery from adequately compensating for the increased metabolic demands induced by excess caloric intake—alcohol in ALD and food in MASLD—resulting in hepatocellular injury. Over four decades ago, Lautt postulated that this ‘oxygen-nutrient mismatch’ could play a role in ALD. We have extended this paradigm to MASLD, theorizing that analogous mechanisms may be involved in both conditions. Evidence that comorbidities, which are associated with recurrent episodes of hypoxemia, such as obstructive sleep apnea (OSA), exacerbate MASLD progression, supports this. ALD is less strongly linked to metabolic syndrome than MASLD. This may be due to inherent differences in hepatic substrate processing. Carbohydrates, lipids, and proteins undergo diverse and flexible cytosolic metabolic pathways, especially under metabolic stress. In contrast, hepatic ethanol metabolism is predominantly linear and obligately oxidative, providing limited metabolic adaptability. Future perspectives could focus on rectifying the imbalance between hepatic oxygen delivery and nutrient availability. This might be accomplished by attenuating hepatic caloric excess using emerging pharmacotherapies for weight reduction, augmenting hepatic oxygenation through hyperbaric oxygen therapy, or increasing hepatic arterial blood flow with agents such as obeticholic acid. Furthermore, enhancement of hepatic basal metabolic activity with thyroid hormone receptor-β agonists, like resmiritom may confer similar therapeutic effects.

## 1. Historical Perspective

Nonalcoholic fatty liver disease (NAFLD/MASLD) is a relatively recent focus in liver research. The specific term, non-alcoholic steatohepatitis (NASH/MASH) was introduced in 1980 by Ludwig and colleagues at the Mayo Clinic [1], while non-alcoholic fatty liver disease, NAFLD, was coined later in 1986 by Schaffner and Thaler [2].

MASLD describes a condition in which the liver shows changes that are virtually identical to those seen in alcoholic liver disease, including lobular hepatitis, focal necrosis with mixed inflammatory infiltrates, Mallory bodies and fibrosis. The key difference is that patients with MASLD do not drink alcohol in excess [3].

Over the past few decades, MASLD has become one of the most common chronic liver diseases globally, tracking with the rise in obesity and metabolic syndrome. It encompasses a spectrum of liver pathology, ranging from simple steatosis (fat accumulation in hepatocytes) to nonalcoholic steatohepatitis (MASH), which is characterized by inflammation, cellular injury, and varying degrees of fibrosis.

Early research often sought to compare the clinical and pathological similarities between NAFLD/MASLD and alcoholic liver disease (ALD). In both conditions, alanine aminotransferase (ALT) and aspartate aminotransferase (AST) are mildly elevated, typically two to three times normal. A key differentiator is the AST: ALT ratio, which is usually less than one with MASLD, but is greater than two in ALD [3]. Increased AST relative to ALT suggests possible mitochondrial damage [4], a topic which will be discussed later.

Given the parallel global rise in obesity and metabolic syndrome, but without a similar increase in alcoholic liver disease, the research focus has shifted. Clinical scientists began to look more closely at the connection between NAFLD/MASLD and metabolic syndrome, rather than comparing the two conditions.

This change in focus is reflected in the recent proposal to rename NAFLD and NASH to metabolic dysfunction-associated steatotic liver disease, MASLD, and metabolic dysfunction-associated steatohepatitis, MASH [5]. The new names, suggested in 2023, also aimed to move away from any stigma associated with alcohol misuse.

MASLD is now recognized as strongly associated with metabolic syndrome, which includes obesity, insulin resistance or type 2 diabetes mellitus, hypertension and dyslipidemia [6]. As we discuss later, the relationship between ALD and metabolic syndrome is much less close.

## 2. Caloric Intake in ALD and MASLD

Both ALD and NAFLD result from the liver being exposed to more calories than it was designed to manage, and for much longer periods. Alcoholic beverages, especially those made by artificial fermentation, contain far higher levels of alcohol than what naturally occurs in foods. For context, an average adult male would need to consume about seventy kilograms of ethanol, his average body weight, over a decade to develop alcoholic steatohepatitis, ASH.

Gastric alcohol dehydrogenase is an enzyme present in the stomach that is involved in the initial processing of alcohol. Under normal circumstances, it can metabolize only the minute quantities of alcohol that are naturally found in foods such as ripening fruits and vegetables. These trace amounts are easily managed by the enzyme, preventing excess alcohol from reaching the liver.

However, when alcoholic beverages are consumed, the concentration of alcohol is much higher than what the enzyme is equipped to handle. As a result, gastric alcohol dehydrogenase becomes saturated very quickly. Once saturation occurs, the enzyme is no longer effective in protecting the liver from the influx of alcohol. Consequently, the excess alcohol bypasses this initial metabolic barrier and enters the liver.

In cases of obesity, the problem centers around excessive intake of food, particularly ultra-processed foods. Obese individuals consume between 4000 and 6000 kilocalories per day (https://caloriehealthy.com/how-much-calories-does-an-obese-person-eat.html, accessed on 17 November 2025).

For reference, ducks or geese raised for foie gras are typically force-fed similar amounts of food (around 3600 to 4000 kilocalories daily), during the gavage phase (https://foiegras-factsandtruth.com/fattening/the-fattening-phase, accessed on 17 November 2025).

Nutrients, including food and alcohol are absorbed from the intestines and delivered to the liver via the portal vein. This delivery occurs in direct proportion to the amount consumed, with little feedback to regulate or limit the delivery, once the nutrient has been absorbed.

**Figure 1:**
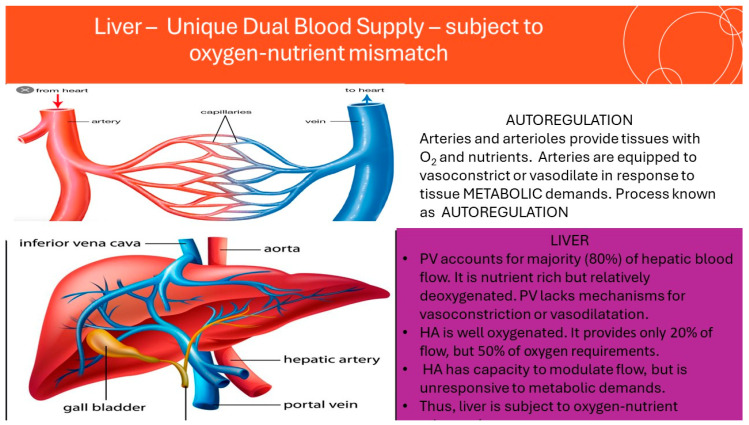
The unique supply and control of blood flow to the Liver.

The unique supply and control of blood flow to the Liver (Figure 1).

The majority of organs and tissues are perfused by arteries that deliver oxygen and nutrients in fixed ratios and can adjust flow to match metabolic demands [7]. However, the liver exhibits notable differences. It is unique in possessing a dual blood supply, with the primary inflow originating from a vein rather than an artery.

Approximately 80% of hepatic blood flow is provided by the portal vein (PV), which drains the intestines. This blood is nutrient-rich and low in oxygen, reflecting its venous origin. Unlike arteries, veins do not possess significant myogenic mechanisms to regulate flow. Consequently, portal vein flow to the liver is unregulated and governed largely by venous pressure.

The remaining 20% of hepatic blood supply is delivered by the hepatic artery (HA). Originating from the systemic circulation, this blood is highly oxygenated. Although the HA contributes only a fifth of the total hepatic blood flow, it supplies at least 50% of the liver’s oxygen requirements [8].

The HA contains myogenic elements that modulate flow via vasoconstriction and vasodilation. In organs with a single arterial supply, this would suffice for metabolic regulation; however, in the liver, where 80% of blood arrives via the unregulated PV, the dual supply limits the organ’s ability to precisely match blood flow with metabolic demand.

Regulation of hepatic blood flow is further distinguished by its scale and significance. Nearly a quarter of the cardiac output flows through the liver. Ensuring this high-volume flow is critical for maintaining cardiovascular and circulatory stability, taking precedence over many other hepatic homeostatic functions.

**Figure 2:**
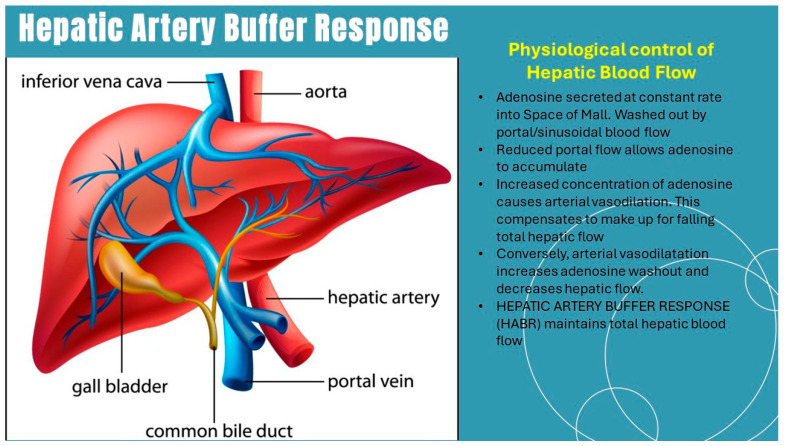
Hepatic artery buffer response.

Perhaps, because of this vital role, the liver has evolved a unique, specialized regulatory mechanism. This involves an interplay between portal blood flow and HA tone. The inverse relationship between HA and PV flow rates was first noted by Burton-Opitz in 1911 [9] and more fully described and termed hepatic arterial buffering response, HABR by Lautt [10,11]. The mechanism of the HABR operates via adenosine washout [12]. Briefly, when flow through the hepatic sinusoids decreases, adenosine accumulates and induces vasodilation of the HA. This increases HA flow to compensate for reduced PV flow, thus stabilizing total hepatic perfusion. As a result, overall hepatic blood flow is maintained independently of the liver’s immediate oxygen or nutrient demands (Figure 2).

## 3. The Concept of Oxygen-Nutrient Mismatch in ALD and MASLD

Unlike most organs and tissues, the liver does not intrinsically regulate its own oxygen or nutrient delivery as may be required by transient metabolic demands. Instead, as stated above, its primary focus is on maintaining a stable overall hepatic blood flow. This approach could result in a discordance between oxygen supply and nutrient influx, particularly when nutrients are presented in excess, predisposing hepatic tissue to hypoxia and subsequent hepatocellular injury.

The hypothesis that an oxygen-nutrient mismatch underlies certain forms of liver pathology was initially articulated by Lautt [13]. Lautt postulated that in alcoholic steatohepatitis, increased ethanol supply from chronic alcohol consumption may exceed the oxygen supply via the hepatic artery, thereby resulting in tissue hypoxia and cellular damage.

We have proposed that a parallel mechanism occurs in non-alcoholic steatohepatitis (NASH), or metabolic dysfunction-associated steatotic liver disease (MASLD), wherein excessive nutrient influx—rather than alcohol—surpasses the hepatic artery’s oxygen delivery, leading to hypoxic injury [14]. This hypothesis could account for several other observations associated with MASLD.

For example, progression of MASLD is accelerated in patients with comorbid conditions characterized by intermittent hypoxia, such as obstructive sleep apnea.

Also, the flow of oxygenated blood across the hepatic acinus from the portal triad to the central vein, establishes an oxygen gradient that is highest in zone 1 and lowest in zone 3. In both alcoholic liver disease (ALD) and MASLD, maximal parenchymal injury occurs in zone 3, where oxygen concentration is lowest, implicating hypoxia in the pathogenic mechanism.

Clinical studies closely associate the progression of fibrosis in NASH with chronic obstructive sleep apnea and episodic hypoxia [15,16,17] further implicating hypoxia in its pathogenesis.

Finally, experimental data from animal models and clinical studies in humans consistently demonstrate a link between hepatic steatosis, fibrosis, and recurrent episodes of hypoxia [15,16,17,18,19,20,21,22,23]. Treatment targeting intermittent hypoxia in patients with NASH has been shown to ameliorate disease severity, further supporting a causal relationship [24,25,26,27]. Thus, although much of the evidence linking hypoxia with MASLD is qualitative, the qualitative data are fairly exhaustive.

## 4. How Ethanol Metabolism Differs from Macronutrient Metabolism in the Liver

The principal clinical and pathological distinction between alcohol-associated liver disease (ALD) and metabolic dysfunction-associated steatotic liver disease (MASLD)—beyond the absence of significant alcohol intake and the frequent presence of obesity in MASLD—relates to the differential elevations of aspartate aminotransferase (AST) and alanine aminotransferase (ALT). Both conditions typically present with mild elevations of ALT and AST, ranging from two to three times the upper limit of normal. However, ALD is characterized by a disproportionately greater increase in AST relative to ALT. In MASLD (formerly NAFLD), the AST:ALT ratio is usually less than one, whereas in ALD it often exceeds two [3]. This pattern is attributed to the primarily mitochondrial localization of AST in hepatocytes; thus, a predominant increase in AST over ALT in serum, likely implicates mitochondrial injury in ALD [4].

The metabolic processing of ethanol differs fundamentally from that of macronutrients. Unlike carbohydrates, lipids, and proteins, which can be stored post-absorption as glycogen, triglycerides, or within protein reserves, ethanol metabolism is not subject to regulatory storage mechanisms. Ethanol cannot be stored and is instead metabolized immediately, often at the expense of other metabolic processes.

Ethanol catabolism, particularly via the microsomal ethanol oxidizing system (MEOS), generates substantial amounts of reactive oxygen species (ROS). The central enzyme in this pathway, cytochrome P450 2E1 (CYP2E1), is upregulated with chronic alcohol exposure and utilizes oxygen inefficiently, resulting in increased ROS production. These ROS inflict oxidative damage on mitochondrial enzymes, proteins, and DNA. Mitochondrial DNA (mtDNA) is notably more vulnerable to oxidative injury, due to its lack of histone protection. The ensuing mitochondrial damage induces apoptotic cell death, which, due to the nature of apoptosis, causes relatively limited release of intracellular enzymes. Consequently, serum AST concentrations in ALD rarely exceed 300 IU/L.

In contrast, the metabolism of carbohydrates, fats, and proteins involves a variety of interrelated cytosolic pathways and generates far lower quantities of ROS. The induction of MASLD generally requires chronic consumption of supraphysiological amounts of macronutrients relative to caloric needs, often leading to obesity. For alcohol-induced liver injury, the approximate threshold for ALD development is the ingestion of around 70 kg of ethanol over a decade for an average 70 kg male. By comparison, the development of MASLD in an obese individual necessitates the consumption of several times the average body weight in macronutrients over the same period.

## 5. Treatment of MASLD Based on Re-Balancing the Oxygen-Nutrient Equation

### Diet, Exercise

For years, dietary modification and physical activity have formed the basis of treatment for NASH/MASLD. Although it is tempting to attribute the benefits of these interventions to a reduction in total caloric intake, evidence suggests the picture is more complex. Altering macronutrient composition, for example, by adopting a higher-protein or Mediterranean-style diet in place of a high-carbohydrate one—can improve liver histology, even when total calories are unchanged [28,29].

The quality of calories is also critical: limiting fructose and saturated fat intake yields particular benefit [30,31,32].This suggests that distinct metabolic pathways are involved in hepatic steatosis, inflammation, and fibrosis depending on both the type of nutrients consumed and the mode of exercise performed.

Ultra-processed foods (UPFs) worsen NASH through mechanisms beyond simple caloric excess. Their rapid digestibility leads to quick surges in blood glucose and lipids, taxing hepatic metabolic systems and stimulating de novo lipogenesis. Additionally, UPFs are rich in additives such as emulsifiers, preservatives, and artificial sweeteners, all of which can compromise gut barrier function [33,34,35].This increased intestinal permeability (“leaky gut”) allows endotoxins like LPS to reach the liver, driving hepatic inflammation—a central process in NASH pathogenesis.

Exercise, even in the absence of dietary changes, has been shown to reduce liver fat and lower ALT levels [36,37,38]. While these effects may partly reflect changes in net energy balance, regular aerobic or resistance training can induce a caloric deficit even without a reduction in food intake.

Insulin resistance offers a unifying explanation for some of the beneficial effects of diet and exercise that extend beyond changes in calorie consumption. Rapid absorption of refined carbohydrates and fats heightens postprandial insulin responses, exacerbating systemic insulin resistance. This, in turn, impairs hepatic lipid metabolism, promoting steatosis and inflammation. Achieving a 5–10% reduction in body weight is associated with improved insulin sensitivity, which correlates with decreased hepatic steatosis, inflammation, and slower fibrosis progression [39].

## 6. Effect of Weight Loss Medications

Caloric restriction and reduced macronutrient intake are established lifestyle interventions for the management of NASH/MASLD, contributing to clinically significant weight loss. The use of anti-obesity medications (AOMs) in this context introduces additional complexity, given the altered hepatic metabolism in MASLD, as well as ongoing concerns regarding drug safety and potential hepatotoxicity.

Novel agents such as glucagon-like peptide-1 (GLP-1) receptor agonists, dual GLP-1/glucose-dependent insulinotropic polypeptide (GIP) agonists, and triple agonists targeting GLP-1, GIP, and glucagon receptors have demonstrated favorable metabolic profiles.

Among these, GLP-1 receptor agonists—including liraglutide and semaglutide—have consistently been associated with clinically beneficial effects including reductions in hepatic steatosis, improvement in serum liver enzymes, and attenuation of fibrosis progression.

Of note, semaglutide, initially approved for obesity in 2017, recently received regulatory approval for the treatment of MASH in adults with moderate-to-advanced hepatic fibrosis [40,41]. Tirzepatide, a dual GLP-1/GIP agonist, has shown superior weight loss effects compared to GLP-1 receptor agonist monotherapy, with emerging but still limited data on hepatic outcomes in MASLD/MASH.

The triple agonist retatrutide has induced the most pronounced metabolic improvements observed to date. The drug enhances glycemic control, significantly reduces body weight and improves insulin sensitivity. Given the effects on weight loss and insulin sensitivity, dramatic effects on liver histology might be anticipated. However, these effects on liver histology have yet to be elucidated.

Other AOMs, including bupropion-naltrexone and phentermine-topiramate, should be prescribed with caution due to concerns over hepatotoxic potential.

It is important to recognize that advanced MASLD may significantly alter drug pharmacokinetics, necessitating individualized treatment regimens and close monitoring [42,43,44].

## 7. Influence of Drugs Modulating Hepatic Blood Flow

Metabolic dysfunction-associated steatohepatitis (MASH), previously termed non-alcoholic steatohepatitis (NASH), is a progressive form of steatotic liver disease (SLD). MASH has emerged as a significant public health concern, given its increasing prevalence, unpredictable and often accelerated course, and ultimate progression to either decompensated liver failure or hepatocellular carcinoma (HCC).

Pathogenesis is complex with strong ethnic influences and genetic predispositions. The complex pathophysiology involves insulin resistance, lipotoxicity, oxidative stress, and chronic inflammation.

Although, this would appear to offer multiple targets for therapeutic intervention, pharmacotherapy remains elusive. While lifestyle changes remain fundamental, their limitations in achieving sustained improvements highlight the need for effective pharmacological and interventional therapies.

Current pharmacotherapeutic strategies target various pathogenic pathways, including the use of farnesoid X receptor (FXR) agonists. FXR has emerged as a central therapeutic target in NASH/MASLD and obeticholic acid (OCA), an FXR agonist, has shown efficacy in clinical trials. However, the occurrence of adverse events such as severe pruritus has thus far prevented its regulatory approval for NASH.

Preclinical studies, including the isolated perfused porcine liver model, have shown that obeticholic acid increases hepatic artery (HA) flow in a dose-dependent fashion, while concurrently reducing portal vein (PV) flow. HA flow rate increased by up to 9.9 ± 8.9% at maximal OCA dose. PV flow fell simultaneously by 19.0 ± 16.0% [14]. Enhancement of HA flow and increasing hepatic oxygen delivery provides a plausible mechanistic explanation for the drug’s beneficial effects in NASH.

Concomitant reduction in portal flow, mediated by the hepatic arterial buffer response (HABR), results in a concomitant decrease in portal pressure—an effect with potential therapeutic benefit in chronic liver disease.

## 8. Potential Impact of Non-Selective Beta-Blockers

Non-selective beta-blockers (NSBBs), such as propranolol [45] and carvedilol [46] remain the standard therapeutic approach for managing portal hypertension. Their primary mechanism for reducing portal pressure has been generally attributed to a decrease in cardiac output via beta-1 adrenergic receptor blockade and a reduction in splanchnic blood flow through beta-2 antagonism [47]. An alternative mechanism, as proposed in our current hypothesis, suggests NSBBs may directly induce vasodilation of the hepatic artery, thereby activating the hepatic arterial buffer response (HABR) and consequently reducing portal vein flow. However, this hypothesis remains to be tested.

Long-term NSBB therapy in patients with stable cirrhosis has been associated with a lower incidence of adverse outcomes—including death, hepatocellular carcinoma, and liver transplantation—even after adjusting for baseline disease severity [48]. In intention-to-treat analyses, carvedilol provided a notable survival benefit, with median survival reaching 7.8 years, compared to 4.2 years in patients treated with variceal band ligation (VBL) (*p* = 0.03). Carvedilol thus confers a significant survival advantage for individuals with cirrhosis and portal hypertension when compared to VBL [49]. This survival advantage, previously unexplained, may reflect enhanced hepatic oxygenation mediated by NSBB administration.

## 9. Enhancing Hepatic Basal Metabolism

Resmetirom is an orally administered, selective agonist of the thyroid hormone receptor-beta (THR-β). Its clinical efficacy derives from its capacity to mimic thyroid hormone activity specifically within hepatic tissue. This selectivity is achieved through hepatic uptake via the OATP1B1 transporter and preferential activation of THR-β over THR-α [50]. Given that THR-β receptors are predominantly expressed in hepatocytes, resmetirom induces a localized, liver-specific hyperthyroid state, thereby minimizing the off-target, systemic side effects typical of conventional thyroid hormone therapies—such as cardiotoxicity and adverse skeletal effects mediated by THR-α [51]. In March 2024, the FDA approved resmetirom for adults with moderate to advanced liver fibrosis secondary to MASH, provided there is no cirrhosis [52,53]. This regulatory decision was underpinned by data from the MAESTRO clinical program, which comprises multiple trials, designed to evaluate the safety and efficacy of resmetirom across various MASH patient subgroups [54].

THR-β is a member of the nuclear hormone receptor superfamily and exerts its effects by binding to thyroid response elements (TREs) within DNA. Upon T3 binding, THR-β1 promotes the transcription of genes involved in key hepatic processes, including cholesterol and lipid metabolism, mitochondrial biogenesis and function, hepatocyte proliferation, and carbohydrate metabolism.

Elevated thyroid hormone levels are known to augment mitochondrial oxygen consumption, stimulate mitochondrial biogenesis, and modulate the expression of proteins governing mitochondrial dynamics (fusion and fission) [55].

Clinical investigations have demonstrated that Resmetirom can improve hepatic histological features, optimize lipid profiles, and induce MASH resolution, all without exacerbating hepatic fibrosis [56].

## 10. Conclusions

Metabolic dysfunction-associated steatotic liver disease (MASLD) is a relatively recent focus in liver research, in which the liver shows changes that are virtually identical to alcoholic liver disease, except that the patients do not consume excess amounts of alcohol. The lack of an acceptable mechanism for its pathogenesis has greatly hampered the search for treatment.

We suggest the oxygen-nutrient mismatch concept could underlie the pathogenesis of both conditions. The stronger link between MASLD and metabolic syndrome—compared to the association between ALD and metabolic syndrome—could be due to differences in how the body metabolizes alcohol versus carbohydrates.

## 11. Directions for Future Research

The pathogenesis of MASLD is intricately layered, shaped by both genetic background and ethnic variation. The major gene polymorphisms influencing MASLD progression include PNPLA3, TM6SF2, GCKR, MBOAT7 and HSD17B13. The ethnic variations in these genes, especially among Hispanic, European and Asian populations, modulate disease risk and severity. Insulin resistance, lipotoxicity, oxidative stress, and persistent inflammation remain core elements within its multifaceted physiological landscape. None of these gene polymorphisms have been shown to directly influence oxygen-nutrient mismatch.

We propose that both ALD and MASLD originate from a fundamental disparity between oxygen supply and nutrient load to the liver. ALD’s weaker association with metabolic syndrome reflects its simpler metabolic handling—ethanol is rapidly metabolized rather than stored—conversely, carbohydrates, lipids, and proteins undergo a complex web of cytosolic metabolic pathways. The connection between MASLD and metabolic syndrome likely reflects the availability of alternative, non-oxidative metabolic routes. Current therapeutic approaches have targeted components of these metabolic networks, but results have been modest. Shifting the therapeutic emphasis toward rectifying the oxygen-nutrient imbalance may offer a more promising solution.

Managing the hepatic delivery of nutrients and ethanol remains a cornerstone of clinical care for this imbalance [40,41]. The introduction of novel anti-obesity medications is poised to enhance our capacity to address this supply-side challenge.

Thyromimetic agents promote the oxidation of surplus calories by selectively mimicking thyroid hormone activity within hepatic tissue, underpinning their therapeutic benefit.

Pharmacologic modulation of hepatic arterial flow presents another potential intervention for both alcoholic and non-alcoholic steatohepatitis. Obeticholic acid, for instance, is hypothesized to act via this mechanism [14]. There is also interest in exploring non-selective beta-blockers for their possible ability to induce hepatic arterial vasodilation.

Hyperbaric oxygen therapy (HBOT) increases the amount of oxygen delivered to the liver and therefore should be carefully examined as a potential strategy to address the mismatch between oxygen and nutrient supply. Notably, HBOT has been linked to episodes of hypoglycemia in diabetic patients receiving treatment for wound infections. This side effect may be due to increased glucose consumption under high oxygen conditions [57].
